# Neuroinflammation Upregulated Neuronal Toll-Like Receptors 2 and 4 to Drive Synucleinopathy in Neurodegeneration

**DOI:** 10.3389/fphar.2022.845930

**Published:** 2022-03-24

**Authors:** Lucia Yi-Ru Chung, Yi-Ting Lin, Chi Liu, Yi-Cheng Tai, Han-Yi Lin, Chin-Hsien Lin, Ching-Chow Chen

**Affiliations:** ^1^ Department of Pharmacology, College of Medicine, National Taiwan University, Taipei, Taiwan; ^2^ Department of Neurology, E-Da Hospital, Kaohsiung, Taiwan; ^3^ Department of Neurology, National Taiwan University Hospital, Taipei, Taiwan

**Keywords:** microglia–neuron coculture, neuroinflammation, Toll-like receptors, autophagic flux, synucleinopathy, neurodegeneration

## Abstract

**Background:** Parkinson’s disease (PD) is characterized by intraneuronal α-synuclein aggregation called Lewy bodies and progressive dopaminergic neurodegeneration. Toll-like receptor (TLR) signaling is a major pathway mediating inflammation. The molecular link on how neuroinflammation upregulates neuronal TLRs and induces accumulation of α-synuclein aggregates to drive synucleinopathy remains to be determined.

**Objective:** Despite conditioned medium from microglia and TLR agonists were utilized to study their effects on neuronal cells, a Transwell coculture system, comprising lipopolysaccharide-activated microglia on top and retinoic acid-differentiated SH-SY5Y cells at the bottom more mimicking *in vivo* neuroinflammation, was employed to elucidate the mechanism of activated microglia on neuronal cells.

**Methods:** Genetic variants of TLRs in PD patients were genotyped and the multiplex cytokines, sRAGE, and HMGB1were assessed. A coculture system was employed to measure α-synuclein aggregates and neurite shortening by confocal microscope. The expression of TLR2/4 and autophagy flux was detected by western blot and immunofluorescence.

**Results:** PD patients showed higher plasma levels of proinflammatory cytokines and genetic *TLR4* variant, *c.896 A > G* (p. D299G). Elevated proinflammatory cytokines in coculture medium was also seen. Phosphorylation and aggregation of α-synuclein, shortening of neurite, upregulation of TLR2/4 expression, activation of downstream p38 and JNK, and dampening of autophagic flux were seen in SH-SY5Y cells cocultured with activated microglia. Those were prevented by inhibiting TLR2/4 and p38/JNK signaling.

**Conclusion:** Activated microglia-derived neuroinflammation induced neuronal TLR2/4-p38/JNK activation to perturb autophagy, causing accumulation of α-synuclein aggregates and neurite shortening. Targeting neuronal TLR2/4 pathway might be a mechanistic-based therapy for neurodegenerative disease, such as PD.

## Introduction

Intraneuronal α-synuclein aggregation called Lewy bodies (LB) and progressive dopaminergic neurodegeneration are the pathological hallmarks of Parkinson’s disease (PD) (McCann et al., 2014). Monomeric form of α-synuclein is soluble, but oligomeric form is aggregated to drive synucleinopathy in neurodegenerative disease, such as PD (Lashuel et al., 2013). Microglia are surveillance cells in central nervous system (CNS) mediating neuroinflammation. The presence of reactive microglia in postmortem substantia nigra of PD patients and in various types of PD models has been shown (McGeer et al., 1988; Ouchi et al., 2005; Tansey et al., 2007). Higher levels of proinflammatory cytokines, including tumor necrosis factor-α (TNF-α), interleukin-1β (IL-1β), interleukin-6 (IL-6), and interferon-gamma (IFN-γ), were found in the cerebrospinal fluids and plasma of PD patients (Muller et al., 1998; Dobbs et al., 1999; Ryul Kim et al., 2018). These indicated that activated microglia mediating inflammatory responses might be involved in the PD pathogenesis (Kim et al., 2013; Changyoun Kim et al., 2018).

Toll-like receptor (TLR) signaling is a major pathway eliciting inflammation, and TLR2 is elevated in the brains of PD (Dzamko et al., 2017; Changyoun Kim et al., 2018). 1-Methyl-4-phenyl-1,2,3,6-tetrahydropyridine (MPTP) neurotoxin was shown to promote neuroinflammation through microglial TLR4 (Mariucci et al., 2018), and oligomeric α-synuclein can activate microglia through TLR2 (Kim et al., 2013). Both TLR2 and TLR4 levels were increased in the blood, gut, and brain tissues of PD patients (Drouin-Ouellet et al., 2014; Dzamko et al., 2017; Perez-Pardo et al., 2019), suggesting an association between TLRs and neuroinflammation in the disease process of PD. However, the molecular link on how neuroinflammation upregulates neuronal TLRs to drive α-synuclein aggregation and neurite shortening still needs further investigation.

The autophagic flux system is a degradative process involving the engulfment of misfolded or aggregated proteins or organelles within autophagosomes by forming autolysosomes to execute the degradation of the engulfing proteins (Yu et al., 2018). Autophagy plays a role in neurodegenerative diseases, including PD (Boland et al., 2008; Higashi et al., 2011; Tanik et al., 2013; Uddin et al., 2018; Croce and Yamamoto, 2019). α-Synuclein is degraded by the autophagy-lysosome pathway (Webb et al., 2003; Boland et al., 2008), and disruption of autophagy flux results in the accumulation of α-synuclein protein (Cuervo et al., 2004; Tanik et al., 2013; Sala et al., 2016).

Since TLRs are expressed in microglia and neurons, whether microglia-derived neuroinflammation upregulated neuronal TLRs to drive α-synuclein aggregation, leading to neurite shortening, was examined by activated microglia cocultured with differentiated dopaminergic neuronal cells. We also analyzed plasma inflammatory cytokines and genetic variants of *TLRs* in a cohort of PD patients and healthy controls.

Genetic variants of *TLRs*, including rs5743611 (p.R80T) in *TLR1*, rs5743708 (p.R753Q) in *TLR2*, rs4986790 (p.D299G) in *TLR4*, intronic and 5′untranslated region variants including rs1927911, rs1927914, and rs10116253 in *TLR4,* and rs5743810 (p.S249P) in *TLR6*, were genotyped, since these variants were reported to increase the risk of PD (Senhaji et al., 2014; Zhao et al., 2015).

To mimic the *in vivo* neuroinflammation, a Transwell coculture system comprising LPS-activated microglial cells on top and retinoic acid-differentiated SH-SY5Y cells, a dopaminergic neuronal cell line, stably expressing GFP-tagged α-synuclein at the bottom was employed (Supplementary Figure S1). Neuroinflammation upregulated neuronal TLR2/4 to aggregate α-synuclein and shorten neurite. TLR2/4-p38/JNK-signaling pathway perturbing the autophagy flux was clarified.

## Materials and Methods

### Part I: Human Study

#### Participants

A total of 1,029 participants, including 516 patients with PD and 513 healthy controls, were recruited from the National Taiwan University Hospital. All PD patients fulfilled the United Kingdom PD Society Brain Bank diagnostic criteria of PD and received regular evaluations of motor and cognitive functions (Hughes et al., 1992). The age and sex were comparable between enrolled PD patients and controls. Participants who have underlying immune-related disorders, including autoimmune diseases, or who have used immune suppressants, or nonsteroid anti-inflammatory drugs within 1 year before the recruitment were excluded. Motor symptom severity was evaluated using the Unified Parkinson’s Disease Rating Scale (UPDRS) motor subscale and Hoehn-and-Yahr staging. All participants provided informed consent before participating the study, and the institutional ethics review board of the National Taiwan University Hospital approved this study (the protocol number is 201703010RINA). All consent was acquired in accordance with the ethical standard in the Declaration of Helsinki.

#### Genotyping of Genetic Variants of Toll-Like Receptors

DNA was obtained from venous blood samples from all 1,029 participants using standard protocols as previously described (Lin et al., 2019). Genetic variants of TLRs, including rs5743611 (p. R80T) in *TLR1* (TaqMan® assay ID: C__27855269_10), rs5743708 (p. R753Q) in *TLR2* (Tagman assay ID: C__27860663_10), and rs4986790 (p. D299G) in *TLR4* (TaqMan® assay ID: C__11722238_20), and intronic and 5’ untranslated region variants including rs1927911, rs1927914, and rs10116253 in *TLR4*, and rs5743810 (p. S249P) in *TLR6* (TaqMan® assay ID: C___1180648_20) were genotyped through real-time polymerase chain reaction (PCR) using TaqMan® Genotyping Assays on a StepOnePlus Real-Time PCR machine (Applied Biosystems).

#### Multiplex Cytokines, Soluble Forms of Receptors for Advanced Glycation End Products and High-Mobility Group Box 1 Assessments

Of the 1,029 participants, plasma samples from 241 participants, including 118 patients with PD and 123 controls, were measured for individual cytokines. Plasma from 10 ml of peripheral venous blood was isolated as previously described (Lin et al., 2019). Cytokine levels, including GM-CSF, IFN-γ, TNF-α, IL-1β, IL-2, IL-4, IL-5, IL-6, IL-12p70, IL-13, and IL-18, were measured in a fixed volume of plasma (25 μl) using a Th1/Th2 Cytokine 11-Plex Human ProcartaPlex™ Panel (Thermo Fisher Scientific Cat. No. EPX110-10810-901) kit following the instructions of the manufacturer. The plasma levels of total soluble forms of receptors for advanced glycation end products (sRAGE) and high-mobility group box 1 (HMGB1) were determined using a commercially available ELISA kit (RAGE, R&D Systems, Minneapolis, MN, USA; HMGB1, IBL, Hamburg, Germany) according to the protocol of the manufacturer. Measurements were performed in duplicate, and the results were averaged.

#### Statistical Analyses

Hardy–Weinberg equilibrium between the expected and observed genotype distributions of PD patients and healthy controls was assessed by Chi-square tests. To assess the relative risk conferred by a particular allele and genotype, odds ratios (ORs) and 95% confidence intervals (CIs) were calculated. A value of *p *< 0.05 was considered statistically significant. All data are presented as the mean ± SD. Variables that followed a Gaussian distribution were compared with two-tailed *t*-tests or analysis of variance. All statistical analyses were performed with Stata 8.0 (StataCorp LP, College Station, TX, USA) software.

### Part II: *In vitro* Coculture of Microglia and SH-SY5Y Cells

#### Cell Lines and Treatment

Cryopreserved and Lenti simian virus-40 (SV40)-immortalized human microglial cells derived from human fetal brain tissue were purchased and cultured on collagen-coated six-well plates (Corning, Corning, NY, USA) in complete DMEM/F-12 medium (Gibco, MD, USA) with 10% FBS (Gibco, MD, USA) according to the instructions of the manufacturer (Innoprot, Derio, Bizkaia, Spain). The human neuroblastoma cell line SH-SY5Y, which exhibits moderate activity of dopamine-β-hydroxylase and tyrosine hydroxylase activity, is widely used for PD research *in vitro* (Krishna et al., 2014). SH-SY5Y cells stably expressing green fluorescent protein (GFP)-tagged α-synuclein were purchased and cultured according to the instructions of the manufacturer (Innoprot, Derio, Bizkaia, Spain), and were differentiated by retinoic acid (RA, Sigma-Aldrich #R2625, St. Louis, MO, USA) as reported (Dzamko et al., 2017) prior to coculture with microglia. Inhibitors of TLRs or p38/JNK were dissolved in DMSO to pretreat differentiated SH-SY5Y cells for 2–4 h prior to coculture. The inhibitors were as follows: TLR1/2 and TLR2/6 (C29, MedChemExpress, NJ, USA), TLR4 (CLI-095), p38 (BIRB-0796, A10148, Adooq Bioscience, CA, USA), and JNK (SP600125, Sigma Chemical Co. Aldrich, MO, USA).

#### Transwell Coculture System

Transwell is a well-characterized 3D model extensively used in cocultures (Renaud and Martinoli, 2016). Microglia and SH-SY5Y cells were cocultured in two chambers: on top were activated microglia in Transwell insert carrying a 0.4-μm semipermeable membrane, and at the bottom were differentiated SH-SY5Y cells (Transwell plate, Corning Incorporated) (Supplementary Figure S1). Prior to coculture, 10 μM retinoic acid were employed to differentiate SH-SY5Y cells plated at a density of 50,000 cells/well on glass coverslips in six-well plates, then washed with PBS and refreshed with 10% FBS-containing DMEM equilibrating for 30 min (Corning CLS3452, Corning, NY, USA). Human microglial cells plated in Transwell insert (1.5 × 10^5^ cells per well) activated by 0.5 or 1 μg/ml of LPS (Sigma Chemical Co. Aldrich, MO, USA) for 24 h were washed by PBS, then placed on top of the differentiated GFP-tagged α-synuclein SH-SY5Y cells (Supplementary Figure S1). After 24 h of coculture, supernatants were collected for measuring cytokines, and SH-SY5Y cells were harvested for further analysis.

#### Real-Time Live Cell Confocal Imaging

Live cell confocal imaging of SH-SY5Y cells was performed using an inverted confocal laser scanning microscope (LSM780; Carl Zeiss microimaging, Inc.) equipped with an incubation system and a Plan-Apochromat 20x/0.8M27 dry immersion objective. Live imaging was performed under an incubation system at 37°C and 5% CO_2_, and two to five images (×20) per sample were taken using the phase-contrast and green fluorescence mode. For assessment of neurite length in SH-SY5Y cells, phase-contrast live cell imaging was used. The image scale was converted from pixel units into micrometers (µm) using ImageJ software (version 1.41, Rasband WS, ImageJ, National Institutes of Health, Bethesda, MD, USA). The length of neurites was traced and measured from the distal end of the neuron growth cone to the tip of the neurite as previously described (Lin et al., 2016). A total of 80–100 neurites were analyzed per group. The somatic GFP-tagged α-synuclein punctate signal represented α-synuclein aggregations from individual cells. A total of 100–150 cells were analyzed per group.

#### Immunofluorescence Labeling Analysis

SH-SY5Y cells stably expressing GFP-tagged α-synuclein were placed at a density of 50,000 cells/well in six-well plates, grown on glass coverslips, and then fixed in 4% PFA (Sigma Chemical Co. Aldrich, St. Louis, MO, USA). Cells were then incubated in phosphate-buffered saline (PBS) containing 0.2% Triton X-100, 1% BSA, and perspective antibodies at 4°C overnight, followed by secondary antibody incubation at room temperature for 30–60 min. After rinsing, the glass coverslips were mounted using ProLong Gold Antifade with DAPI (Invitrogen, CA, USA).

#### Image Analysis

Fluorescence intensity was measured using corrected total cell fluorescence (CTCF) and analyzed by ImageJ software (version 1.41; National Institute of Health, Bethesda, Maryland, United States) and calculated using the following formula: CTCF = Integrated Density—(Area of selected cell × Mean fluorescence of background signal). Co-localization was analyzed using IMARIS x64 v9.5.4 software (BITPLANE, Oxford Instruments) (Costes et al., 2004) provided by the First Core of College of Medicine, National Taiwan University.

#### Multiplex Cytokine Measurements

The analysis of 23 cytokines and chemokines, including IL-1α, IL-1β, IL-2, IL-3, IL-4, IL-5, IL-6, IL-7, IL-8, IL-10, IL-13, IL-15, IFN-γ, TNF-α, TNF-β, TGF-β1, MCP-1, MCP-2, MCP-3, G-CSF, GM-CSF, MIG, and RANTES was performed on coculture medium with microglial cells before and after stimulation by LPS using a cytokine antibody array (catalog number: ab133996, Abcam Cambridge, MA, USA) following the instructions of the manufacturer.

#### RNA Isolation and Quantitative Real-Time Polymerase Chain Reaction

The mRNA levels of IL-1β, IL-12, and IL-18 in microglial cells before and after stimulation with LPS were determined by quantitative RT-PCR. Total RNA from human microglial cells was extracted using TRIzol (Invitrogen, Carlsbad, CA, USA) and reverse-transcribed into cDNA using SuperScriptTM III reverse transcriptase (Invitrogen, Carlsbad, CA, USA). Quantitative RT-PCR was performed on a cDNA amount equivalent to 100 ng of total RNA using SYBR Green PCR master mix (Applied Biosystems, Foster City, CA, USA) and was performed on ABI Prism 7,900 (Applied Biosystems, Foster City, CA, USA). The housekeeping gene is GAPDH.

#### Western Blot Analysis

Cocultured SH-SY5Y cells were collected by trypsinization from a Transwell system, and cell lysates as well as western blot analysis were conducted as previously described (Lin et al., 2015). Antibodies and qPCR primer sequences are listed in Supplementary Materials.

#### Statistical Analyses

Statistical analysis for more than two groups, one-way ANOVA multiple comparison was used. Between the two groups, *t*-test was used. All data are presented as the mean ± SEM. Statistical significance was defined as a *p*-value of less than 0.05. All statistical analyses were performed with Stata 8.0 (StataCorp LP, College Station, TX, USA) software.

## Results

### Plasma Levels of Cytokines are Increased in Parkinson’s Disease Patients

Patients with PD and healthy controls were recruited from National Taiwan University Hospital, and their genetic variants for *TLRs* and plasma levels of cytokines were examined (Supplementary Table S1 and Table 1). In the multiple comparison shown in Table 1, the cutoff *p*-value for the statistical significance was 0.05/13 = 0.0038. In this regard, plasma level IL-2, IL-4, IL-18, IFN-γ, TNF-α, and GM-CSF were increased in the PD group compared with those of controls (Table 1), while that of IL-1β (*p* = 0.01) and IL-12p70 (*p* = 0.01) between PD and controls did not reach the statistical difference. In the study cohort, most of the genetic variants of *TLR1*, *TLR2*, *TLR4*, and *TLR6* were wild type, except rs4986790 (c.896A > G p. D 299G) in *TLR4* and rs5743810 (c.745 T > C S249P) in *TLR6* (Supplementary Table S2). There was a modest difference in the genotype distribution of PD patients compared with the age- and sex-matched control subgroup (*p* = 0.04) in the nonsynonymous variant of *TLR4* (c.896A > G p. D 299G; rs4986790), and the frequency of the G allele was higher in the PD group (odds ratio (OR) = 3.03, 95% CI j = 1.03–9.45, *p* = 0.04) (Supplementary Table S2). These results suggest that the systemic inflammatory response was increased in PD patients and that *TLR-4* genetic variants might increase the risk of PD.

**TABLE 1:** Plasma levels of cytokines, high-mobility group box 1 (HMGB1) , and soluble forms of receptors for advanced glycation end products (sRAGE) in patients and age-/sex-matched controls.

	Controls n = 123	PD patients n = 118	*p*-Value^a^
IL-1β (pg/ml)	2.45 ± 1.46	2.81 ± 1.36	*p* = 0.01
IL-2 (pg/ml)	6.74 ± 3.34	9.44 ± 5.00	*p < *0.01^b^
IL-4 (pg/ml)	13.04 ± 5.68	7.54 ± 4.42	*p* = 0.04
IL-5 (pg/ml)	8.48 ± 4.12	6.16 ± 3.29	*p* = 0.09
IL-6 (pg/ml)	13.67 ± 6.19	13.90 ± 8.51	*p* = 0.62
IL-12p70 (pg/ml)	8.34 ± 4.23	10.81 ± 6.01	*p* = 0.01
IL-13 (pg/ml)	2.22 ± 1.15	1.93 ± 1.47	*p* = 0.18
IL-18 (pg/ml)	65.17 ± 38.61	156.36 ± 85.56	*p < *0.01^b^
IFN-r (pg/ml)	28.42 ± 13.45	38.61 ± 15.33	*p < *0.01^b^
TNF-α (pg/ml)	3.91 ± 2.27	5.37 ± 2.91	*p < *0.01^b^
GM-CSF (pg/ml)	7.35 ± 5.29	10.15 ± 5.27	*p < *0.01^b^
HMGB1	2.45 ± 1.86	2.54 ± 1.91	*p* = 0.83
sRAGE	1,028.92 ± 481.53	906.75 ± 385.56	*p* = 0.06

Note. Data are expressed as mean ± SD.

a *p *< 0.05.

b *p *< 0.01. *p*-Values were obtained from comparisons of individual characteristics between two groups with two-tailed *t*-test followed by Bonferroni’s correction. For variables that did not display a normal distribution, data were compared with the Mann–Whitney test, the nonparametric equivalent of the independent sample *t*-test. IL-1β, interleukin-1β; IL-6, interleukin-6; IFN-γ, interferon-gamma; TNF-α, tumor necrosis factor-α.

### Activated Microglia Cocultured With Differentiated SH-SY5Y Cells Increases Proinflammatory Cytokines and Leads to Neuronal α-Synuclein Aggregation and Neurite Shortening

**FIGURE 1:**
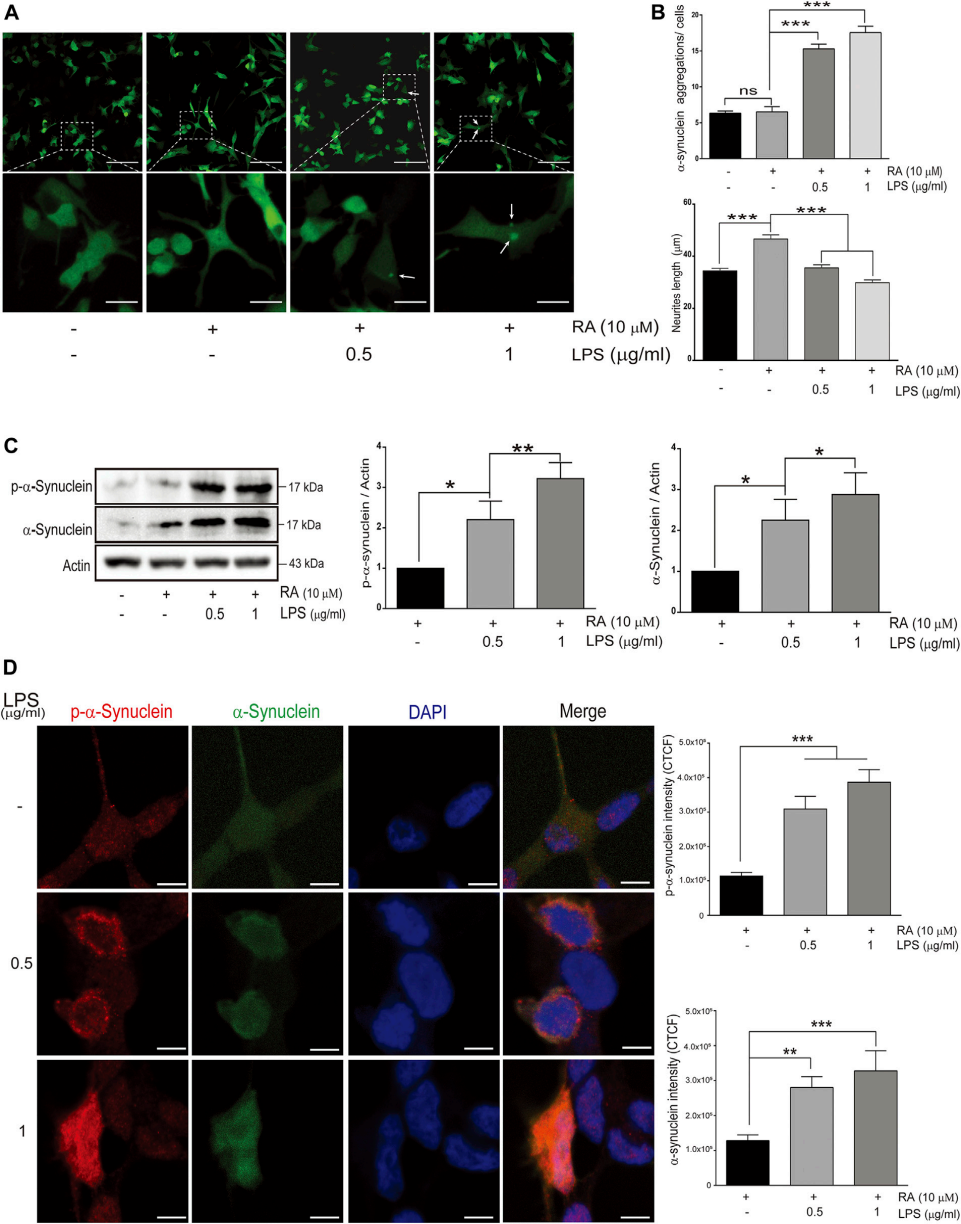
Alpha-synuclein aggregation in SH-SY5Ycells cocultured with lipopolysaccharide (LPS)-activated HMC for 24 h **(A)** Confocal live-cell imaging of cocultured SH-SY5Y cells with 0.5 and 1 μg/ml LPS-activated HMC for 24 h; scale bar represents 10 μm (above) and 5 μm (below). **(B)** Quantification of α-synuclein aggregations (green dots) and length of SH-SY5Y neurites (neurites from 100 cells per group were analyzed in phase images) from cocultured SH-SY5Y cells with activated microglia (*N = *4). ns, not significant, ***p < 0.001 by one-way multiple comparison ANOVA. **(C)** The expression of phosphorylated α-synuclein and α-synuclein in SH-SY5Y cells were analyzed by western blot of SH-SY5Y cells, and lysate was collected after coculture with 0.5 and 1 μg/ml LPS-activated HMC for 24 h. Actin was used as a loading control (*N* = 4). All bars stand for mean ± SEM; ns, not significant, *p < 0.05, **p < 0.01 by one-way multiple comparison ANOVA. **(D)** Cocultured SH-SY5Y cells were fixed in paraformaldehyde and stained with p-α-synuclein (red). Immunofluorescence was quantified using corrected total cell fluorescence (CTCF) (*N* = 3), scale bar represents 10 μm. All bars stand for mean ± SEM; ns, not significant, **p < 0.01 by one-way multiple comparison ANOVA.

To explore the link between neuroinflammation and neuronal TLRs in the neurodegeneration, an *in vitro* microglial–neuronal coculture system was employed. Microglial cells are the major orchestrator of the brain’s inflammatory response, and LPS-induced microglial activation mimics the process of neuroinflammation (Batista et al., 2019). Abnormal accumulation of neuronal α-synuclein and neurite shortening are the main pathological hallmarks of neuroinflammation, such as in PD (Spillantini et al., 1997). Compared with SH-SY5Y cells cocultured with non-activated microglia, those cocultured with activated-microglia were found in increasing the number of GFP-tagged α-synuclein aggregations (Figure 1A, third and fourth panels; 1B, upper: third and fourth columns) (Figure 1B, upper: cocultured with non-activated HMCs, non-differentiated SH-SY5Y: 6.34 ± 0.29; differentiated SH-SY5Y: 6.52 ± 0.71 with *p* = 0.84; cocultured with 0.5 μg/ml LPS-activated HMCs, differentiated SH-SY5Y: 15.3 ± 0.7 with *p * 0.0001; 1.0 μg/ml LPS-activated HMCs, differentiated SH-SY5Y: 17.6 ± 0.9 with *p < *0.0001), as well as shortened neurites (Figure 1B, lower: cocultured with non-activated HMCs, non-differentiated SH-SY5Y: 34.39 ± 0.95, differentiated SH-SY5Y: 46.61 ± 1.56 μm with *p *< 0.0001; cocultured with 0.5 μg/ml LPS-activated HMCs, differentiated SH-SY5Y: 35.54 ± 1.19 μm with *p *< 0.0001; 1.0 μg/ml LPS-activated HMCs, differentiated SH-SY5Y: 29.84 ± 1.07 μm with *p *< 0.0001).

Posttranslational modifications of α-synuclein elicit self-aggregation, and phosphorylation of α-synuclein, especially on serine 129, accounts for more than 90% of aggregated α-synuclein in Lewy bodies (Fujiwara et al., 2002; Anderson et al., 2006). Western blot and immunofluorescence showed an increase in the total form and p-Ser129-α-synuclein with aggregated punctate distributions in SH-SY5Y cells cocultured with activated microglia (Figures 1C,D) (Figure 1C: western blot of p-α-synuclein: basal versus 0.5 μg/ml LPS-activated HMCs, *p* = 0.037; 1 μg/ml LPS, *p* = 0.003. Western blot of α-synuclein: basal versus 0.5 μg/ml LPS-activated HMCs, *p* = 0.047; 1 μg/ml LPS, *p* = 0.017) (Figure 1D: Fluorescence of p-α-synuclein: basal versus 0.5 μg/ml and 1 μg/ml LPS-activated HMCs, *p *< 0.0001. Fluorescence of α-synuclein: basal versus 0.5 μg/ml LPS, *p* = 0.0072; 1 μg/ml LPS, *p* = 0.0005).

**FIGURE 2:**
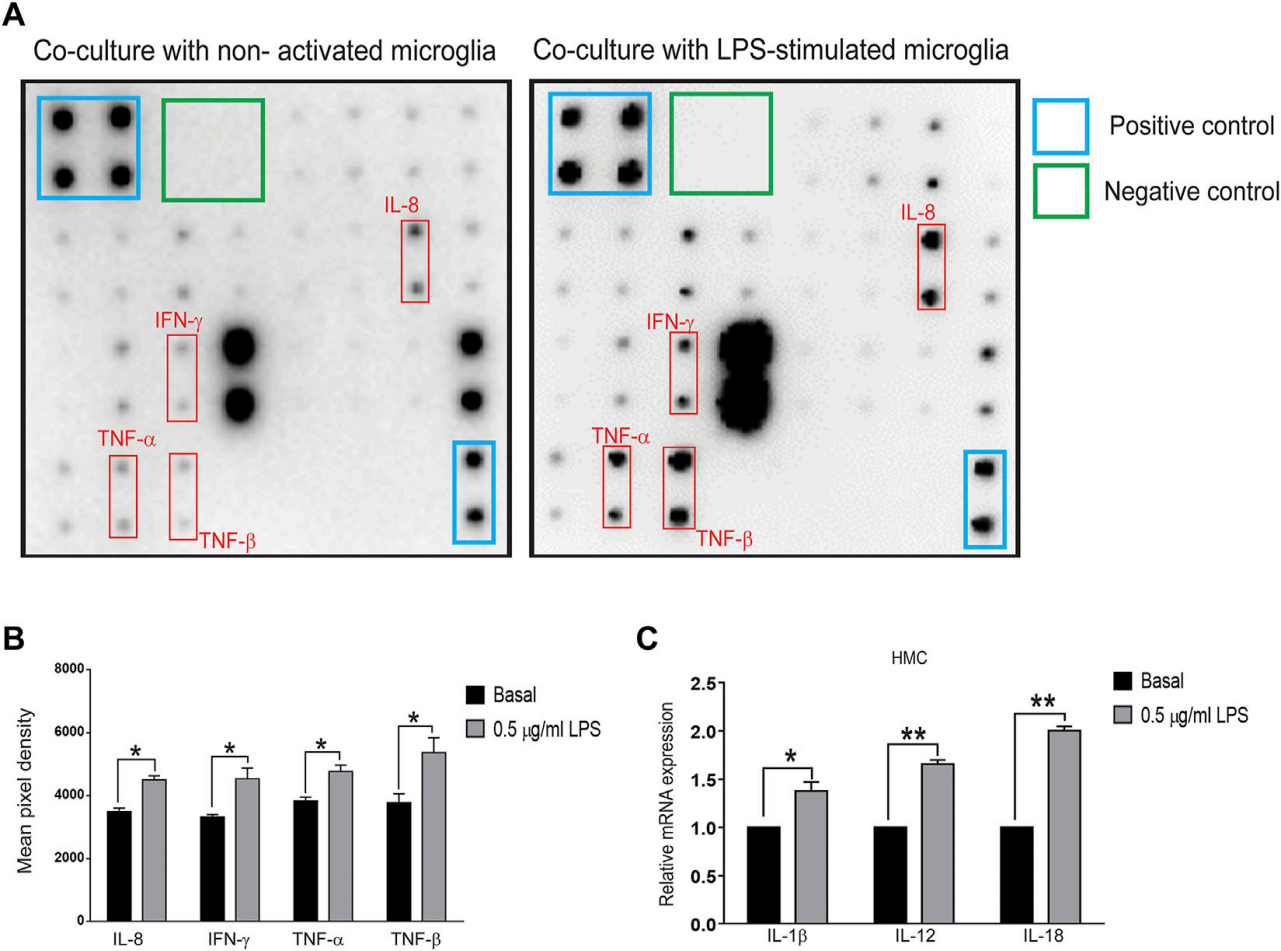
Cytokine array and qPCR of inflammatory molecules in the cocultured system of SH-SY5Y and LPS-treated HMC cells **(A,B)** Supernatants were collected after the coculture system without LPS-treated (basal) or 0.5 μg/ml LPS-treated HMC with SH-SY5Y cells after 24 h. **(A)** Blots and **(B)** quantification of interleukin-8 (IL-8), interferon-gamma (IFN-γ), tumor necrosis factor-α (TNF-α), TNF-β (*N* = 3), and **(C)** mRNA levels of interleukin-1β (IL-1β), IL-12, and IL-18 in cocultured HMCs measured by quantitative polymerase chain reaction (qPCR) (*N* = 3). All bars represent mean ± SEM; ns, not significant, *p < 0.05, **p < 0.01 versus basal by paired *t*-test.

To further determine whether inflammatory proteins are responsible for α-synuclein aggregation in neurons, a multiplex protein array was employed to examine the expression profiles of cytokines in the medium collected from the Transwell coculture system. Upregulation of IL-8 (*p* = 0.0304), IFN-γ (*p* = 0.031), TNF-α (*p* = 0.028), and TNF-β (*p* = 0.045) was observed (Figures 2A,B), and qRT-PCR analysis showed increases in IL-1β (*p* = 0.042), IL-12 (*p* = 0.0014) and IL-18 (*p* = 0.002) in microglial cells (Figure 2C). These data implied that activated microglia-mediated neuroinflammation might promote neuronal α-synuclein pathology, recapitulating the pathology of neurodegeneration, such as PD.

### Neuronal Toll-Like Receptors 2/4 was Elevated and the p38/JNK Pathway was Activated in SH-SY5Y Cells Cocultured With LPS-Activated Microglia

**FIGURE 3:**
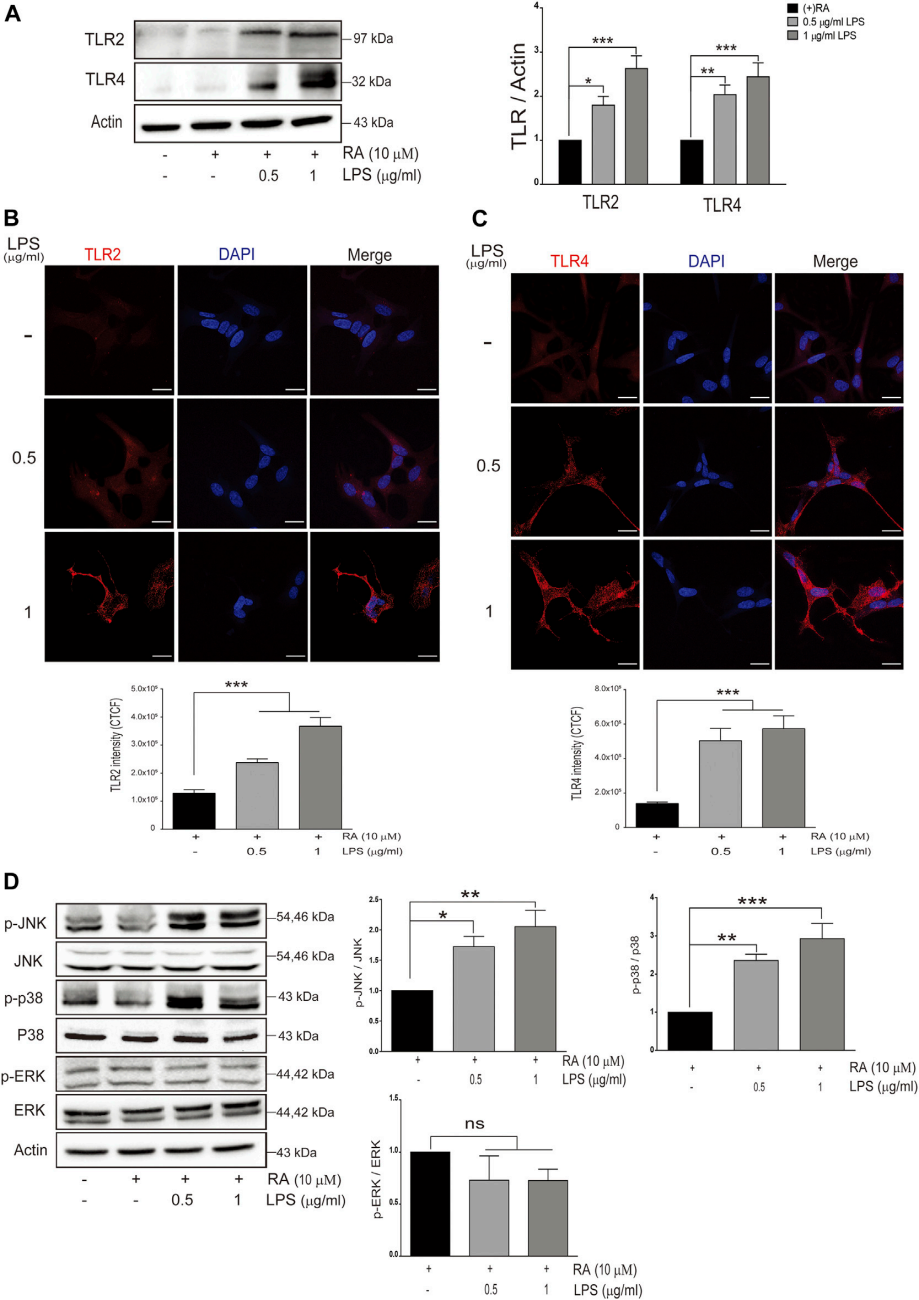
Neuronal Toll-like receptors (TLRs) are induced with LPS-activated HMC and downstream signaling. **(A)** SH-SY5Y cell lysates were collected after coculture with 0.5 and 1 μg/ml LPS-activated HMC for 24 h, and the expressions of Toll-like receptor 2 (TLR2) and Toll-like receptor 4 (TLR4) in SH-SY5Y were analyzed by western blot. Actin was used as a loading control (*N* = 4). All bars stand for mean ± SEM, *p < 0.05, **p < 0.01, ***p < 0.001 by one-way multiple comparison ANOVA. **(B,C)** SH-SY5Y cells cocultured with 0.5 and 1 μg/ml LPS-activated HMC for 24 h, and cocultured SH-SY5Y cells were fixed in paraformaldehyde and stained with **(B)** TLR2 (red) and **(C)** TLR4 (red) (*N* = 3). Intensity was measured using corrected total cell fluorescence (CTCF). Scale bar represents 10 μm. All bars stand for mean ± SEM; ***p < 0.001 by one-way multiple comparison ANOVA. **(D)** Expression levels of p-JNK, p-p38, and p-ERK were analyzed by western blot. Actin was used as a loading control (*N* = 4). All bars stand for mean ± SEM, *p < 0.05, **p < 0.01, ***p < 0.001 by one-way multiple comparison ANOVA.

We next examined the expression levels of TLRs in differentiated SH-SY5Y cells cocultured with LPS-activated microglia. Among them, TLR2 and TLR4 in neurons were increased in variable extents (Figure 3A) (TLR2: basal versus 0.5 μg/ml LPS-activated HMCs, *p* = 0.0382; 1 μg/ml LPS, *p* = 0.0006. TLR4: basal versus 0.5 μg/ml LPS-activated HMCs, *p* = 0.0097; 1 μg/ml LPS, *p* = 0.0004). Immunofluorescence further demonstrated the increased intensity of TLR2 (basal versus 0.5 μg/ml LPS-activated HMCs, *p* = 0.0002; 1 μg/ml LPS, *p *< 0.0001) and TLR4 (basal versus 0.5 and 1 μg/ml LPS-activated HMCs, *p *< 0.0001) in SH-SY5Y neuronal cells (Figures 3B,C).

Activation of JNK (p-JNK) and p38 (p-p38) but not ERK (p-ERK) in SH-SY5Y cells was also observed (Figure 3D) (p-JNK: basal versus 0.5 μg/ml LPS-activated HMCs, *p* = 0.04; 1 μg/ml LPS, *p* = 0.005. p-p38: basal versus 0.5 μg/ml LPS-activated HMCs, *p* = 0.0072; 1 μg/ml LPS, *p* = 0.0008. p-ERK: basal versus 0.5 μg/ml LPS-activated HMCs, *p* = 0.37; 1 μg/ml LPS, *p* = 0.36).

### Autophagosome-Lysosome Fusion Was Disrupted in SH-SY5Y Cells Cocultured With Activated Microglia as Well as Concurrence of Pro-caspase-3 Cleavage in Neuronal Cells

**FIGURE 4:**
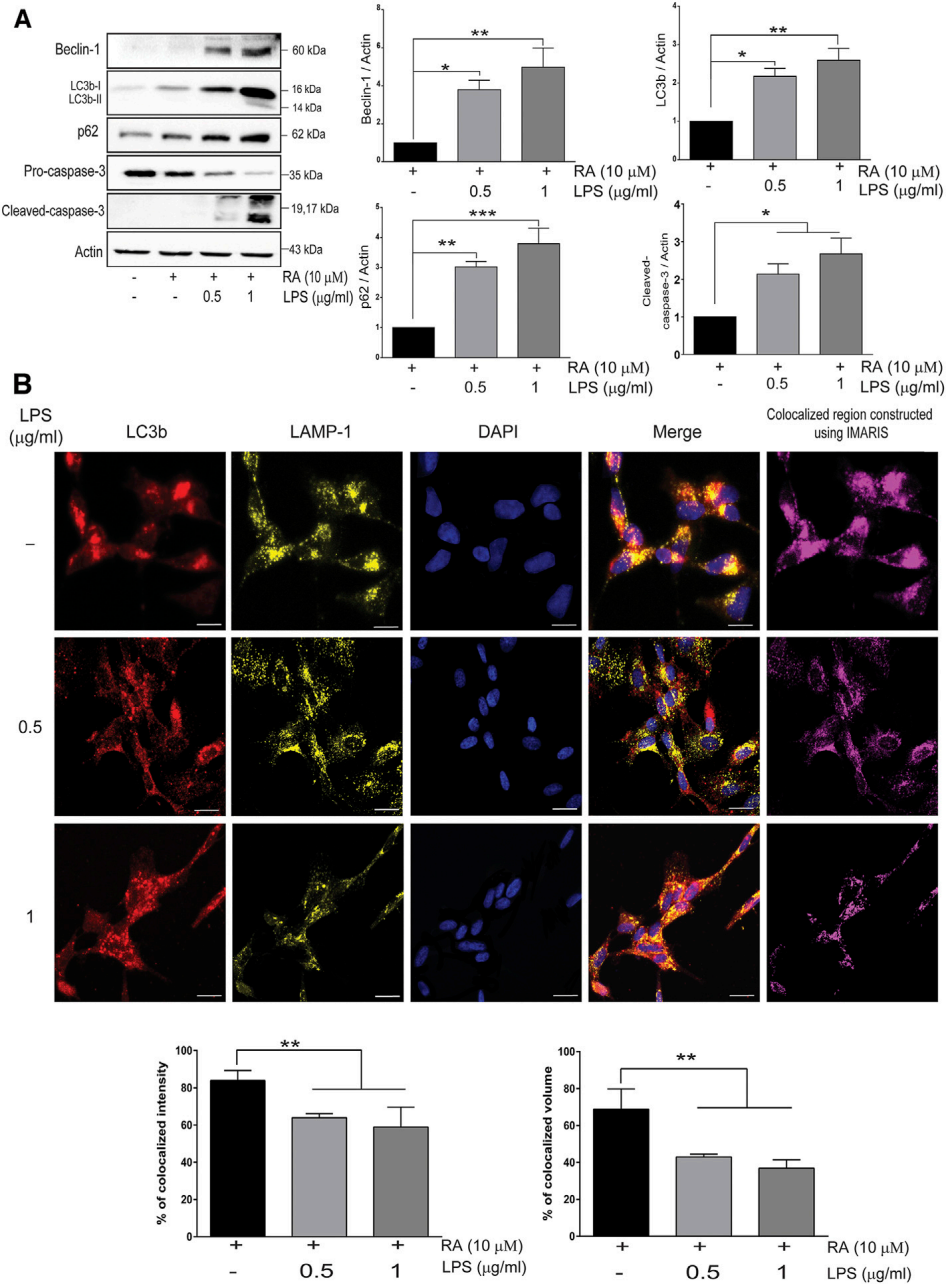
Neuroinflammation obstructs neuronal autolysosome formation in an inflammatory state. **(A)** SH-SY5Y cell lysates were collected after cocultured with 0.5 and 1 μg/ml LPS-activated HMCs for 24 h. The expressions of beclin-1, LC3b, p62, and caspase-3 in SH-SY5Y cells were analyzed by western blot, and actin was used as a loading control. All bars stand for mean ± SEM; ns stands for not significant; *p < 0.05, **p < 0.01, ***p < 0.001 by one-way multiple comparison ANOVA. **(B)** Cocultured SH-SY5Y cells were fixed in paraformaldehyde and stained with LC3b (red), LAMP-1 (yellow), and DAPI (blue) (*N* = 3). Scale bar represents 10 μm; ns, not significant; all bars stand for mean ± SEM; ns stands for not significant; *p < 0.05 by one-way multiple comparison ANOVA.

PD was reported to be associated with lysosomal, proteasomal, and mitochondrial dysfunction (Narendra et al., 2008; Xilouri and Stefanis, 2011; Xilouri et al., 2013), and autophagy-related markers in SH-SY5Y cells cocultured with activated microglia were examined. The autophagosome formation protein Beclin-1, autophagosome membrane protein LC3b-I/II, and autophagy flux marker p62 were increased, as was the cleavage of neuronal pro-caspase-3 (Figure 4A) (Beclin-1: basal versus 0.5 μg/ml LPS-activated HMCs, *p* = 0.038; 1 μg/ml LPS, *p* = 0.008. LC3b: basal versus 0.5 μg/ml LPS-activated HMCs, *p* = 0.015; 1 μg/ml LPS, *p* = 0.003. p62: basal versus 0.5 μg/ml LPS-activated HMCs, *p* = 0.022; 1 μg/ml LPS, *p* = 0.0003. Cleaved caspase-3: basal versus 0.5 μg/ml LPS-activated HMCs, *p* = 0.032; 1 μg/ml LPS, *p* = 0.013). Upregulation of LC3b-I/II indicates autophagosome maturation, whereas upregulation of p62 suggests dampening autophagic flux (Liu et al., 2016). p62 is degraded under normal autophagic flux; however, upregulation is seen in nondegraded autophagosomes (Liu et al., 2016). Accumulation of autophagosome occurs by failing to fuse with lysosome to form autolysosomes or dysfunction of lysosome activity (Darios and Stevanin, 2020). To clarify this, autophagosome marker LC3b labeled with red and lysosome marker LAMP-1 labeled with yellow were employed (Figure 4B). Co-expression of LC3b and LAMP-1 in the cytosol of SH-SY5Y cells was observed when the cells were cocultured with non-activated microglial cells (Figure 4B, first row). However, a decrease in the colocalization quantitated by the IMARIS software was found when cocultured with activated microglial cells (Figure 4B, second and third row). These results indicated that neuroinflammation dampened autolysosome formation, leading to the accumulation of neuronal autophagosomes along with α-synuclein pathology (Figure 1) and caspase-3 activation (Figure 4A). It is probable that upregulated neuronal TLR2 and TLR4 mediated by activated microglia-derived neuroinflammation activate p38/JNK (Figure 3D) to promote autophagosome accumulation as well as α-synuclein phosphorylation and aggregation, resulting in neurite shortening in SH-SY5Y cells.

### Toll-Like Receptor 2/4 Inhibitors Ameliorated α-Synuclein Aggregation and Neurite Shortening by Enhancing Autophagic Flux Through Attenuating p38/JNK Activation

To further examine the role of upregulated neuronal TLR2 and TLR4 in α-synuclein pathology, differentiated SH-SY5Y cells were pretreated with inhibitors of TLR2 or TLR4 before coculture with activated microglia. Both TLR2 and TLR4 inhibitors, C29 and CLI-095, prevented neurite shortenings during neuroinflammation and also reduced α-synuclein aggregation (Supplementary Figure S2). In addition, both inhibitors ameliorated the accumulation of phosphorylated α-synuclein and p62 as well as attenuated the activation of p38/JNK (p-JNK and p-p38, Figures 5A,B) (p-α-synuclein: basal versus 10 μM C29, *p* = 0.39; 50 μM C29 *p* = 0.0036; 1 μg/ml CLI-095, *p* = 0.0011; 5 μg/ml CLI-095, *p* = 0.0017. α-synuclein: basal versus 10 μM C29, 50 μM C29, 1 μg/ml CLI-095, and 5 μg/ml CLI-095, *p *> 0.99. p-JNK: basal versus 10 μM C29, *p* = 0.38; 50 μM C29 *p* = 0.01; 1 μg/ml CLI-095, *p* = 0.17, and 5 μg/ml CLI-095, *p* = 0.007. p-p38: basal versus 10 μM C29, *p* = 0.75; 50 μM C29, *p* = 0.0161; 1 μg/ml CLI-095, *p* = 0.02, and 5 μg/ml CLI-095, *p* = 0.009. LC3b: basal versus 10 μM C29, 50 μM C29, 1 μg/ml CLI-095 and 5 μg/ml CLI-095, *p *> 0.99. p62: basal versus 10 μM C29, *p* = 0.85; 50 μM C29, *p* = 0.0408; 1 μg/ml CLI-095, *p* = 0.0059 and 5 μg/ml CLI-095, *p* = 0.0051).

**FIGURE 5:**
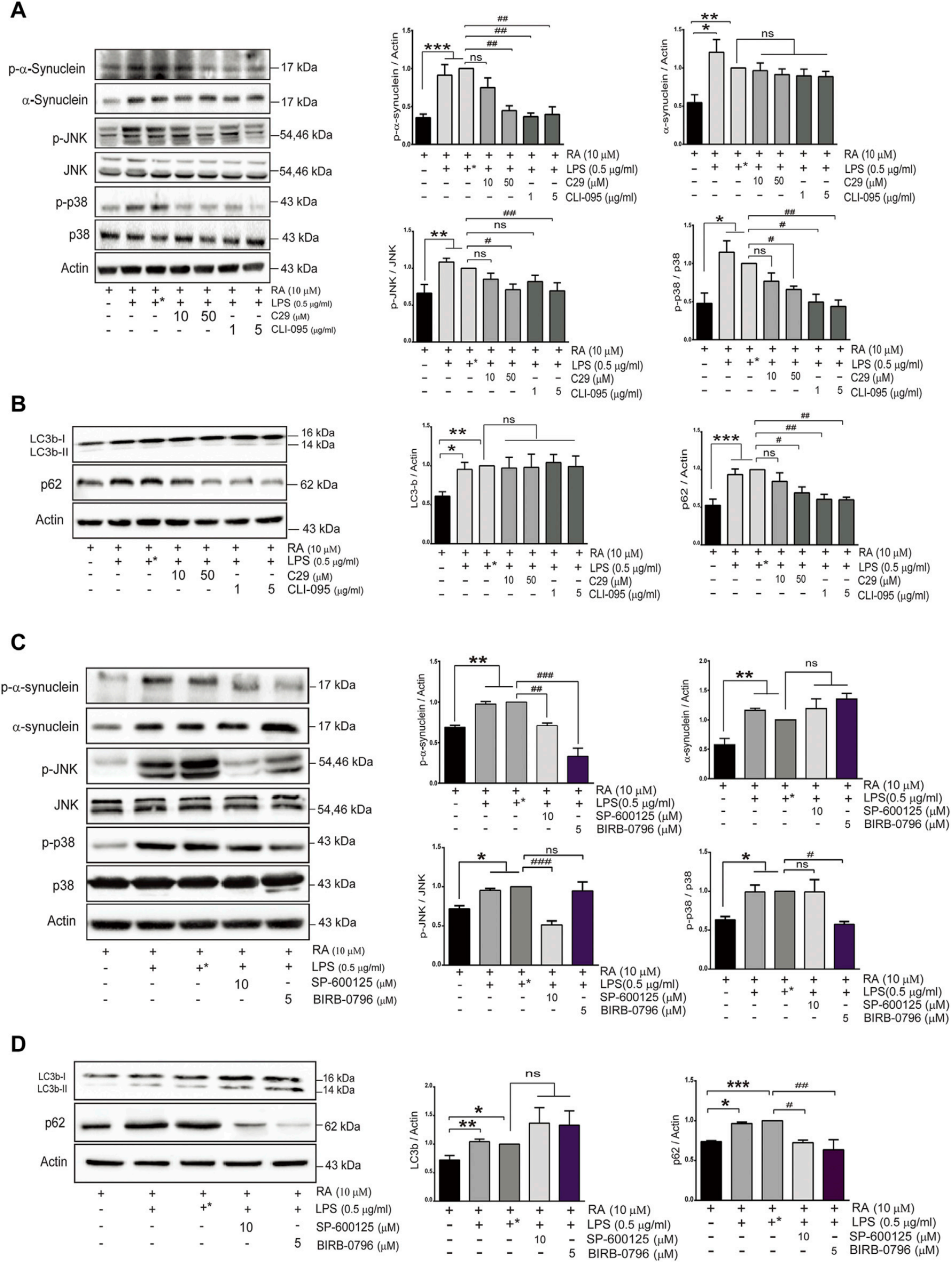
TLR inhibitors mitigate α-synuclein aggregation and improve SH-SY5Y autophagy flux activity in an inflammatory state. SH-SY5Y cells were treated with or without the TLR2 inhibitor (C29; 10 and 50 μM) and the TLR4 inhibitor (CLI-095; 1 and 5 μg/ml) dissolved in DMSO for 4 h prior to coculture with 0.5 μg/ml LPS-treated HMC for 24 h. The expression of **(A)** p-α-synuclein, α-synuclein, p-JNK, JNK, p-p38 and p38, and **(B)** autophagy markers, p62 and LC3b, in cocultured SH-SY5Y cells were analyzed by western blot. Actin was used as a loading control. **(C,D)** SH-SY5Y cells were treated with or without the JNK inhibitor (SP600125; 10 μM) and the p38 inhibitor (BIRB-0796; 5 μM) for 2 h prior to coculture with 0.5 μg/ml of LPS-treated HMC for 24 h. The expression of **(C)** p-α-synuclein, α-synuclein, p-JNK, JNK, p-p38 and p38, and **(D)** autophagy markers, LC3 and p62 were analyzed by western blot. Actin was used as a loading control for cocultured SH-SY5Y cells (*N* = 4). +^*^: represent SH-SY5Y cells were treated with solvent DMSO as inhibitors and then removed and washed before cocultured with LPS-treated HMC. All bars stand for means ± SEM. ns, not significant, * represents *p*-value compared with basal, and # represents *p*-value compared with DMSO-treated SH-SY5Y cells. * or #*p *< 0.05, ** or ##*p *< 0.01, *** or ###*p *< 0.001 by one-way multiple comparison ANOVA.

To further examine whether p38 and JNK activation is involved in the accumulation of phosphorylated α-synuclein and p62 induced by TLR2/4, BIRB-0796 (p38 inhibitor) and SP600125 (JNK inhibitor) were employed and found to prevent neurite shortening as well as reduce α-synuclein aggregation during neuroinflammation (Supplementary Figure S3). In addition, both inhibitors attenuated the levels of p-α-synuclein and p62 (Figures 5C,D) (p-α-synuclein: basal versus 10 μM SP600125 *p* = 0.0086; 5 μM BIRB-0796 *p *< 0.0001. α-synuclein: basal versus 10 μM SP600125, *p* = 0.818; 5 μM BIRB-0796, *p* = 0.124. p-JNK: basal versus 10 μM SP600125, *p* = 0.0007; 5 μM BIRB-0796 *p *> 0.099. p-p38: basal versus 10 μM SP600125, *p *> 0.099; 5 μM BIRB-0796, *p* = 0.0224). These results suggested that inhibition of the TLR2/4-JNK/p38 pathway not only improved autophagic flux to ameliorate α-synuclein phosphorylation and aggregation, but also prevented neurite shortening. Therefore, the TLR2/4-p38/JNK pathway might decrease autophagic flux to enhance α-synuclein aggregation, resulting in neurodegeneration.

## Discussion

**FIGURE 6:**
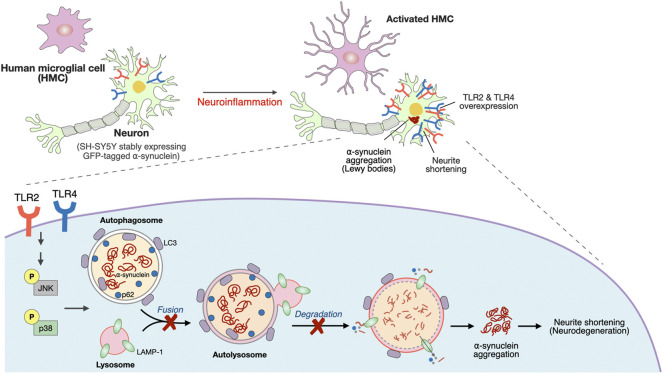
Schematic representation of a potential mechanism that neuroinflammation upregulated neuronal TLR2/4 expression to drive α-synuclein aggregation in neurodegeneration disease, such as Parkinson’s disease (PD).

In this study, *TLR4* genetic variants and an elevation of the systemic inflammatory response in PD patients were revealed. A Transwell coculture system comprising activated microglia and differentiated SH-SY5Y cells, which more mimics the *in vivo* condition is employed to explore the underlying mechanism on how neuroinflammation induced upregulation of neuronal TLRs to drive synucleinopathy. Activated microglia-mediated neuroinflammation upregulated neuronal TLR2 and TLR4 to activate the p38/JNK pathway resulting in the inhibition of autophagic flux. This cascade leads to α-synuclein aggregation and neurite shortening as illustrated in Figure 6.

Postmortem and animal models of PD have revealed an involvement of neuroinflammation in the neurodegeneration of PD (Hirsch and Hunot, 2009; Lin et al., 2019). Current increase in plasma IL-1β, IL-2, TNF-α, and IFN-γ in PD patients is consistent with the previous findings in spinal cerebrospinal fluid and postmortem brain (Nagatsu et al., 2000; Qin et al., 2016). Previous studies have shown that the plasma levels of HMGB1 and sRAGE were increased in patients with neurodegenerative disorders, including PD (Santoro et al., 2016; Yang et al., 2018). However, we failed to observe the difference between the PD and the control groups. Future studies enrolling more participants with a longitudinal study design are needed to clarify their role in the pathogenesis of PD.

Under pathologic conditions, peripheral inflammatory cytokines can cross the blood–brain barrier to induce activation of microglia, resulting in neuroinflammation (Nagatsu et al., 2000; Qin et al., 2016; Ryul Kim et al., 2018; Lin et al., 2019). Upon activation, TLR-overexpressed microglia modulate inflammatory reactions (Kielian, 2006). Among them, TLR2 and TLR4 are responsible for the recognition of bacterial peptidoglycans and LPS, respectively, and both TLR2 and TLR4 are reported to be increased in the blood and brains of PD patients (Drouin-Ouellet et al., 2014; Dzamko et al., 2017). Microglial TLR2/4 can also recognize neuron-released oligomeric α-synuclein to activate microglia and induce neuroinflammation (Fellner et al., 2013; Kim et al., 2013; Rannikko et al., 2015).

Neuronal TLR2 was expressed and activated in differentiated dopaminergic neuron-like SH-SY5Y cells, primary human neural progenitor cells, and mature neurons (Dzamko et al., 2017). In contrast to microglia and astrocytes, the role of neuronal TLR in neuroinflammation and downstream signaling cascades still needs to be investigated (Tang et al., 2007; Rietdijk et al., 2016). The increased TLR2 in postmortem brain neuron of PD patients was confirmed as well as the correlation and localization of TLR2 with the accumulation of pathological α-synuclein (Dzamko et al., 2017). They studied the pathway mediating disease pathogenesis by treating TLR2/TLR1 dimer agonist PAM3CSK4 to RA-differentiated SH-SY5Y cells in the absence of microglia. Although the increase in α-synuclein protein at 3- and 7-day treatments in SH-SY5Y cells was found, the western blots were not consistent (Dzamko et al., 2017). Authors admitted that intrinsic production of inflammatory cytokines by neuronal SH-SY5Y cells was insufficient to promote cell death and suggested that activation of TLR2 on neurons requires amplification of the inflammatory response following the recruitment and activation of microglia (Dzamko et al., 2017). Actually, activated microglia are prevalent in the pathologically effected areas in brains of patients with PD and Alzheimer’s disease (McGeer et al., 1988; Hughes et al., 1992). Our Transwell coculture system comprising microglial cells and differentiated SH-SY5Y cells demonstrated that microglial cells are critical for the expression of neuronal TLR2/TLR4. Only activated microglia mediating neuroinflammation can upregulate neuronal TLR2 and TLR4, while non-activated microglia (in the absence of LPS-activation) fail to exhibit such effects after 24-h coculture. In addition, our human study revealed that PD patients show increased inflammatory responses as well as *TLR4* genetic variant, consistent with the report that a genetic variant of *TLR4* is associated with the risk of PD (Zhao et al., 2015).

TLR activation is reported to inhibit autophagic activity (Oh and Lee, 2014; Kim et al., 2015), and fibrillar α-synuclein was also shown to reduce autophagic flux activity (Tanik et al., 2013). TLR2 ablated autophagy activity *via* accumulation of autophagosomes, and p62 was reported to result in the accumulation οf neuronal α-synuclein (Kim et al., 2015). Our findings further demonstrated that neuroinflammation upregulated TLR2 and TLR4 in SH-SY5Y cells to activate the p38/JNK pathway, leading to the increase in phosphorylation and accumulation of α-synuclein proteins and shortening neurites. Under neuroinflammation, we also found an increase in the autophagy marker, beclin-1, and LC3b-I/II, indicating the formation of autophagosomes. Furthermore, an elevation of p62 parallel with a decrease in the cytosolic co-localization of LC3b-I/II and LAMP-1 were seen (Figures 4A, B), suggesting the disruption of autolysosome formation by dampening the fuse of lysosome with autophagosomes. In the presence of TLR2/4 inhibitors, only p62 but not LC3b (autophagosomes formation) was reduced, indicating that dampened autophagic flux might occur and explain the aggregation of α-synuclein, and neurite shortening in neuronal cells. However, in the absence of microglia, RA-differentiated SH-SY5Y cells upon PAM3CSK4 treatment only showed elevation of p62 but not beclin 1 and LC3 (Dzamko et al., 2017).

We demonstrated that p38 and JNK activations were downstream of TLR2/4 to reduce autophagy. JNK and p38 were reported to be involved in different neurodegenerative diseases through mediating apoptosis (Oh-Hashi et al., 1999). In contrast, pharmacological inhibition of p38 increases autophagy (Boland et al., 2008; Schnoder et al., 2016), and downregulation of p38α induces lysosomal activation to consequently reduce amyloid-β accumulation in an animal model of Alzheimer’s disease (AD) (Schnoder et al., 2016). We found that the specific inhibitor, BIRB-0796, inhibited neuronal p38 activation and decreased p62 level as well as phosphorylated α-synuclein. However, among a collection of small-molecule inhibitors targeting TLR signal transduction pathway, BIRB-0796 had no effect on p62 level despite reducing α-synuclein protein (Dzamko et al., 2017). A recent report also indicated that p38 mediated microglial activation through inhibiting autophagy (He et al., 2018). Consistent with a previous finding that neuronal TLR2 is involved in the inhibition of autophagic activity to accumulate α-synuclein aggregates in neurons (Kim et al., 2015), our findings extend current knowledge to reveal that both neuronal TLR2 and TLR4 act through the p38/JNK pathway to disrupt the formation of autolysosome, and also partly explain the recent report that TLR4 mediates inflammation in the gut and neuronal α-synuclein accumulation in the enteric nervous system (Perez-Pardo et al., 2019).

## Conclusion

Our study demonstrates the role of neuroinflammation-induced upregulation of neuronal TLR2 and TLR4 in α-synuclein pathology, and neurodegeneration through regulating p38/JNK pathway to perturb the autophagy flux (summarized schematically in Figure 6). The TLR2/4 pathway could be a mechanism-based therapeutic strategy for neurodegenerative disease such as PD.

## Data Availability

The original contributions presented in the study are included in the article/Supplementary Material. Further inquiries can be directed to the corresponding authors.

## Abbreviations

PD, Parkinson’s disease; LB, Lewy bodies; LPS, lipopolysaccharide; CNS, central nervous system; UPDRS, Unified Parkinson’s Disease Rating Scale; TLR4, Toll-like receptor 4; TLR2, Toll-like receptor2; TNF-α, tumor necrosis factor-α; IL-1β, interleukin-1β; IL-6, interleukin-6; IFN-γ, interferon-gamma; MPTP, 1-methyl-4-phenyl-1,2,3,6-tetrahydropyridine; RA, retinoic acid.
