# Supplementary material for: Neuroinflammation Upregulated Neuronal Toll-Like Receptors 2 and 4 to Drive Synucleinopathy in Neurodegeneration
Source: Front Pharmacol. 2022 Mar 24;13:845930. doi: 10.3389/fphar.2022.845930 (PMC8987529; doi:10.3389/fphar.2022.845930)
Supplement: Supplementary file 1 [file DataSheet1.docx]

**Supplementary Information**

**Inventory of Supplementary Information**

Supplementary Materials

Supplementary Table 1

Supplementary Table 2

Supplementary Figure S1

Supplementary Figure S2

Supplementary Figure S3

**Supplementary Materials**

**A. Primary antibodies**

| Antibodies | Catalog no. | Brand |
| --- | --- | --- |
| Rabbit anti-phospho SAPK/JNK (Thr183/Tyr185) | 9251S | Cell Signaling Technology, MA, USA |
| Rabbit anti-phospho-p44/42 MAPK (Erk1/2) (Thr202/Tyr204) | 9101S |  |
| Rabbit anti-ERK | 9102S |  |
| Rabbit-anti-phospho-p38 MAPK (Thr180/Tyr182) | 9211S |  |
| Rabbit anti-p38 | 9212S |  |
| Rabbit anti-Caspase-3 | 9662S |  |
| Rabbit anti-α-synuclein | 2642S |  |
| Rabbit anti- IκBα | sc-371 | Santa Cruz, CA, USA |
| Rabbit anti-JNK | sc-474 |  |
| Mouse anti-β-actin | sc-47778 |  |
| Rabbit anti-TLR2 | NB100-56585, NB100-56722 | Novus Biological, CO, USA |
| Rabbit anti-TLR4 (For Immunofluorescence) | NBP-100-56566 |  |
| Rabbit anti-TLR9 | NBP2-24729SS |  |
| Rabbit anti-TLR5 | GTX-31724 | GeneTex Inc, CA, USA |
| Rabbit anti-p62 | GTX-100685 |  |
| Rabbit anti-LC3B | GTX-127375 |  |
| Rabbit anti-phospho-Ser129 α-synuclein | ab51253 | Abcam, MA, USA |
| Rabbit ant-TLR4 (For WB) | H000007099-M02 | Abnova, Taipei, Taiwan |

**B. Secondary antibodies**

| Antibodies | Catalog no. | Brand |
| --- | --- | --- |
| HRP conjugated anti-rabbit | 7074S | Cell Signaling Technology MA, USA |
| HRP conjugated anti-mouse | 7076S |  |
| Alexa Fluor 594-conjugated anti-rabbit antibody | A32740 | Invitrogen |
| Dylight 650-conjugated anti-mouse antibody | A90-116D5 | Bethyl laboratories, TX, USA |
| Dylight 550-conjugated anti-rabbit antibody | A120-101D3 |  |

**C. qPCR primer sequences**

| Target | Primer sequence | |
| --- | --- | --- |
| IL-1β | F' | 5′-ATGATGGCTTATTACAGTGGCAA-3′ |
|  | R' | 5'-GTCGGAGATTCGTAGCTGGA-3′ |
| IL-18 | F' | 5'-GATAGCCAGCCTAGAGGTATGG-3' |
|  | R' | 5'-CCTTGATGTTATCAGGAGGATTCA-3' |
| GAPDH | F' | 5'-ACAGTCAGCCGCATCTTC-3' |
|  | R' | 5'-GCCCAATACGACCAAATCC-3' |
| IL-12p70 | HP100118 (Sino biological) | |

**Supplementary Table**

**Supplementary Table 1.** Basic characteristics of all study participants

|  | **Plasma cytokine and biomarkers assay** | | ***P* value** | **Genetic analysis of *TLR* genes** | | ***P* value** |
| --- | --- | --- | --- | --- | --- | --- |
|  | **Controls (n=123)** | **PD patients (n=118)** |  | **Controls (n=513)** | **PD patients (n=516)** |  |
| ***Clinical features*** |  |  |  |  |  |  |
| Age (years) | 63.29$\pm$10.82 | 64.33$\pm$8.21 | 0.75 | 65.25$\pm$10.23 | 67.11$\pm$12.1 | 0.12 |
| Male sex (%) | 64 (52.0%) | 62 (52.5%) | 0.94 | 254 (49.5%) | 260 (50.4%) | 0.78 |
| Disease duration (years) | N.A. | 6.2$\pm$7.5 | - | N.A. | 9.5$\pm$7.3 | - |
| Hoehn-Yahr stage (on) | N.A. | 2.2$\pm$0.8 | - | N.A. | 3.4$\pm$0.7 | - |
| UPDRS part III (on) | N.A. | 16.8$\pm$7.9 | - | N.A. | 31.6$\pm$11.7 | - |
| MMSE | N.A. | 28.1$\pm$0.8 | - | N.A. | 22.7$\pm$5.7 | -. |

Data are expressed as mean±standard deviation, except for sex as numbers (proportion). N.A.: not available. Abbreviations: TLR, toll-like receptors; UPDRS, unified Parkinson’s disease rating scale; MMSE, mini-mental state exam.

**Supplementary Table 2.** Distribution of genotypes and estimated odds ratio of TLRs genetic variants in relation to risk of PD

|  | **Controls**  **n=513** | **PD patients**  **n=516** | **OR (95% CI)** | ***P* value ^a^** |
| --- | --- | --- | --- | --- |
| ***TLR4 c.896 A>G D299G (rs4986790)*** |  |  |  |  |
| *AA* | 509 (99.2%) | 504 (97.7%) |  |  |
| *AG* | 4 (0.8%) | 12 (2.3%) |  |  |
| *GG* | 0 (0) | 0 (0) |  |  |
| *G vs. A allele* |  |  | 3.03 (1.03-9.45) | *P*=0.04* |
| ***TLR6 c.745 T>C S249P (rs5743810)*** |  |  |  |  |
| *TT* | 511 (99.6%) | 516 (100.0%) |  |  |
| *TC* | 2 (0.4%) | 0 (0) |  |  |
| *CC* | 0 (0) | 0 (0) |  |  |
| *C vs. T allele* |  |  | 0.99 (0.98-1.41) | *P*=0.98 |

PD, Parkinson’s disease; OR, odds ratio; CI, confidence interval. ^a^ Fisher’s exact test was applied.

**Supplementary Figures**

**
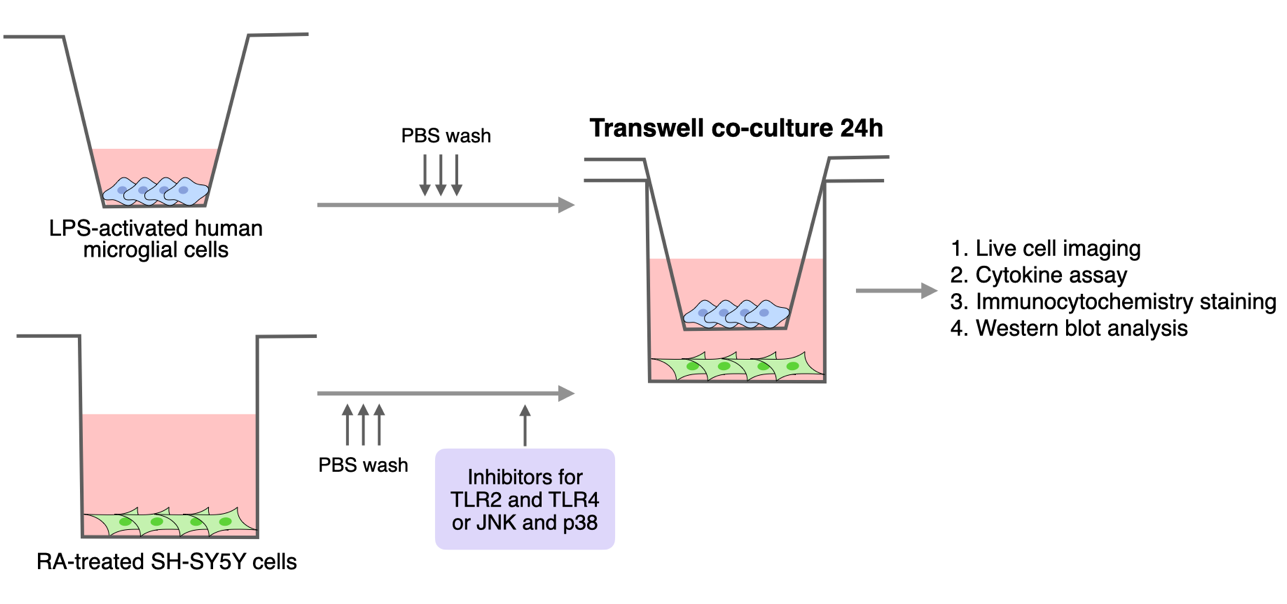
**

**Supplementary Figure S1** Schematic diagram of the co-culture procedure.


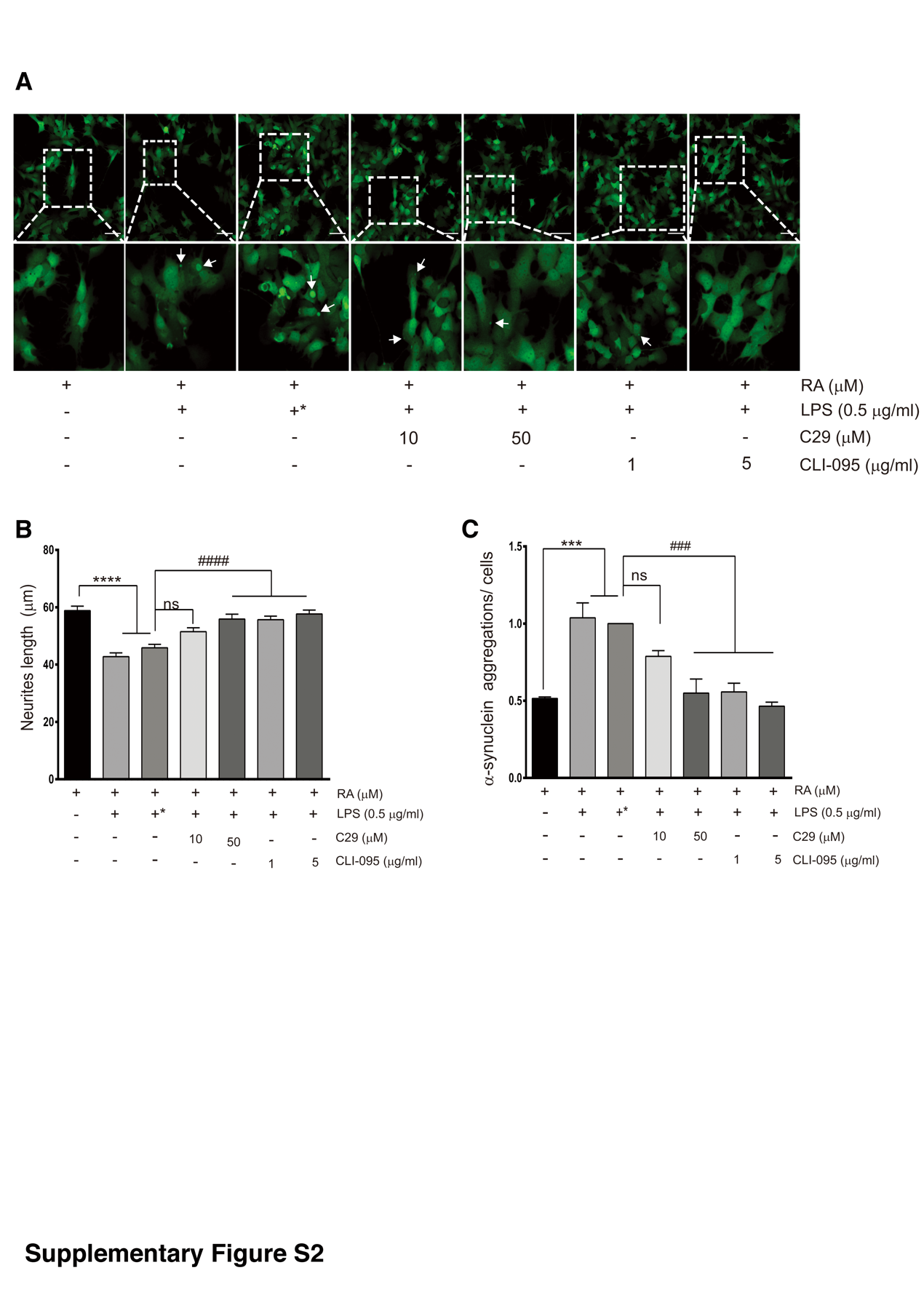


**Supplementary Figure S2** Pre-treatment of SH-SY5Y cells with TLR2 and TLR4 inhibitors protected neurites during inflammation. SH-SY5Y cells were treated with or without the TLR2 inhibitor (C29; 10 and 50 μM) and the TLR4 inhibitor (CLI-095; 1 and 5 μg/ml) dissolved in DMSO for 4 hours prior to coculture with 0.5 μg/ml LPS-treated HMC for 24 hours. **(A)** Confocal live-cell imaging of cocultured SH-SY5Y cells, scale bar represents 10 μm (above). **(B)** Length of SH-SY5Y neurites (neurites were calculated for <100 cells per group in phase images) (*N*=3). **(C)** Quantification of α-synuclein aggregations (green dots) (*N*=3). **+^*^:** SH-SY5Y cells were treated with DMSO, then removed and washed to co-culture with LPS-treated HMC. All bars represent mean ± SEM, * represents *P* value compared to basal, and # represents *P* value compared to DMSO-treated SH-SY5Y. ns, not significant, **P*<0.05, ** *P*<0.01 by one-way multiple comparison ANOVA.

**
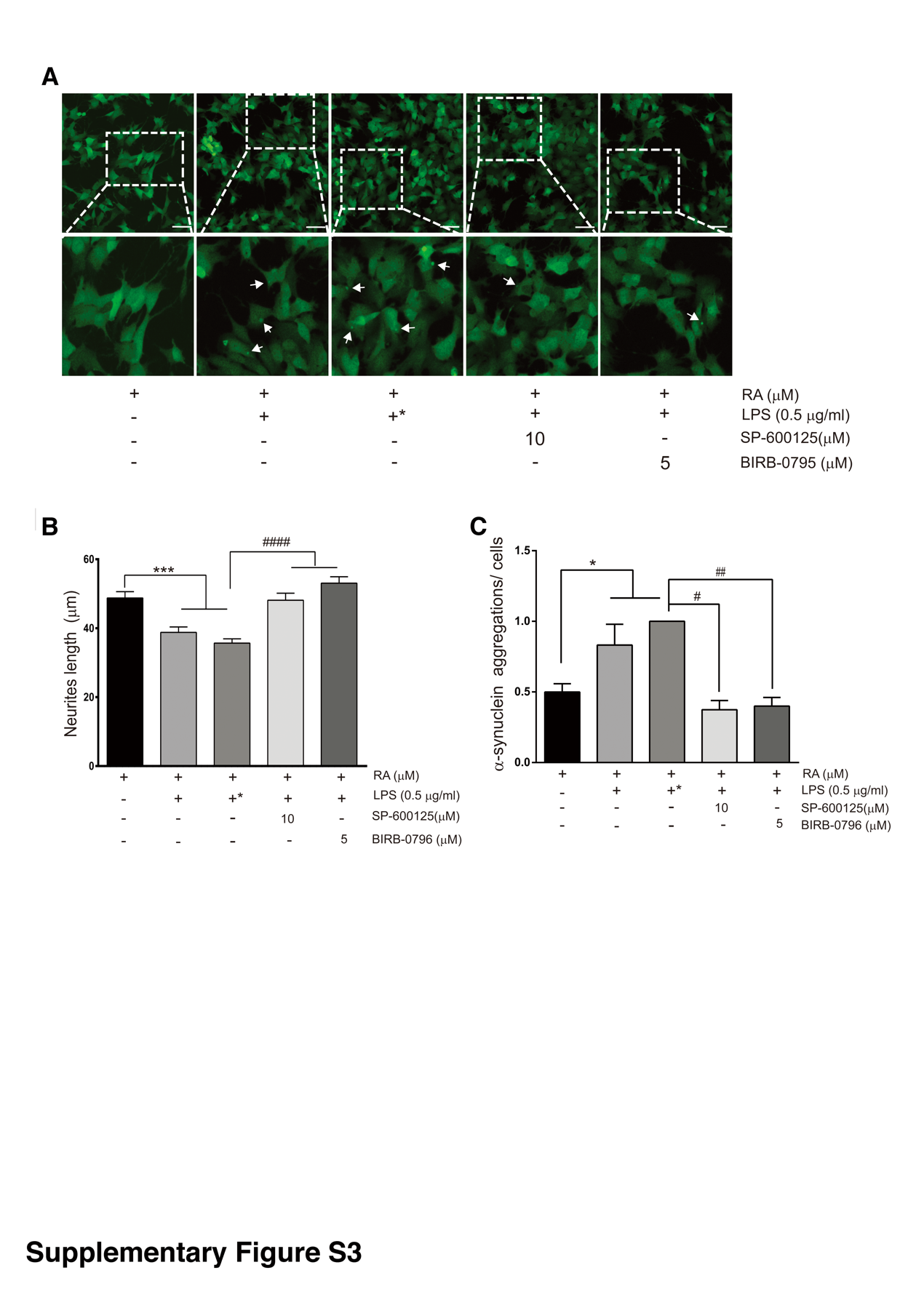
**

**Supplementary Figure S3** Pre-treatment of SH-SY5Y cells with JNK and p38 inhibitors protect neurites from shrinkage. SH-SY5Y cells were treated with or without the JNK inhibitor (SP600125; 10 μM) and the p38 inhibitor (BIRB-0796; 5 μM) dissolved in DMSO for 2 hours prior to coculture with 0.5 μg/ml LPS-treated HMC for 24 hours. **(A)** Confocal live-cell imaging of cocultured SH-SY5Y, scale bar represents 10 μm (above). **(B)** Length of SH-SY5Y neurites (neurites were calculated for <100 cells per group in phase images) (*N*=3). **(C)** Quantification of α-synuclein aggregations (green dots) from cocultured SH-SY5Y cells with activated microglia (*N*=3). **+^*^:** SH-SY5Y cells were treated with solvent DMSO, then removed and washed to co-culture with LPS-treated HMC. All bars represent mean ± SEM. * represents *P* value compared to basal, and # represents *P* value compared to DMSO-treated SH-SY5Y cells. ns, not significant, **P*<0.05, ** *P*<0.01 by one-way multiple comparison ANOVA.
